# Changes in oxidative patterns during dormancy break by warm and cold stratification in seeds of an edible fruit tree

**DOI:** 10.1093/aobpla/plw024

**Published:** 2016-05-06

**Authors:** Dilinuer Shalimu, Jia Sun, Carol C. Baskin, Jerry M. Baskin, Liwei Sun, Yujun Liu

**Affiliations:** ^1^National Engineering Laboratory for Tree Breeding, College of Biological Sciences and Biotechnology, Beijing Forestry University, Beijing 100083, China; ^2^Department of Biology, University of Kentucky, Lexington, KY 40506, USA; ^3^Department of Plant and Soil Sciences, University of Kentucky, Lexington, KY 40546, USA

**Keywords:** H_2_O_2_, lipid peroxidation, NO, physiological dormancy, pomegranate seeds, protein carbonylation, scavenging enzymes

## Abstract

The transition from seed dormancy to germination is triggered by environmental factors, and in pomegranate (*Punica granatum*) seeds higher germination percentages are achieved by warm + cold stratification rather than by cold stratification alone. Our objective was to define the pattern of internal oxidative changes in pomegranate seeds as dormancy was being broken by warm + cold stratification and by cold stratification alone. Embryos isolated from seeds after 1–42 days of warm stratification, after 56 days of warm stratification + 7, 28 or 56 days of cold stratification, and after 1–84 days of cold stratification alone, were used in biochemical tests. Hydrogen peroxide (H_2_O_2_), nitric oxide (NO), proline, lipid peroxidation, protein carbonylation, and activities of the scavenging enzymes superoxide dismutase (SOD), hydrogen peroxide enzyme and peroxidase in the embryos were assessed by colorimetric methods. Our results indicated that warm + cold stratification had a stronger dormancy-breaking effect than cold stratification (85% versus 50% germination), which may be attributed to a higher yield of H_2_O_2_, NO, lipid peroxidation and protein carbonylation in warm + cold stratification. Furthermore, warm + cold stratification-induced H_2_O_2_ change led to greater changes (elevation followed by attenuation) in activities of the scavenging enzymes than that induced by cold stratification alone. These results indicated that restriction of the level of reactive oxygen species change within a positive and safe range by such enzymes promoted seed germination. In addition, a relatively strong elevation of proline during warm + cold stratification also contributed to dormancy breakage and subsequent germination. In conclusion, the strong dormancy alleviating effect of warm + cold stratification on pomegranate seeds may be attributed to the corresponding active oxidative change via H_2_O_2_, NO, proline, malondialdehyde, protein carbonylation and scavenging enzymes.

## Introduction

Germination *sensu stricto* includes all the events from the start of water uptake (imbibition) by dry quiescent seeds to elongation of the embryonic axis and protrusion of the radicle (Bewley 1997). Dormancy is an innate feature of seeds that enables them to regulate timing of germinating to occur when habitat conditions, particularly water and temperature, are favourable for successful seedling establishment and completion of the plant life cycle (Finch-Savage and Leubner-Metzger 2006). Seed dormancy is influenced by both environmental and endogenous factors (Graeber *et al.* 2012), and it is an important component of plant fitness (Donohue *et al.* 2005; Huang *et al.* 2010).

Five classes of seed dormancy are recognized among plant species: physiological dormancy, morphological dormancy, morphophysiological dormancy, physical dormancy and combinational dormancy (Baskin and Baskin 2004; Finch-Savage and Leubner-Metzger 2006). Physiological dormancy is the most common class of dormancy, and it can be further divided into deep, intermediate and non-deep levels, of which the non-deep level accounts for a substantial proportion of all dormant seeds (Graeber *et al.* 2012; Baskin and Baskin 2014). Generally, non-deep physiological dormancy can be broken by: (i) dry storage of seeds (after-ripening) under ambient laboratory conditions, (ii) warm (moist at ≥15 °C) or cold (moist at 0–10 ºC) stratification or (iii) warm stratification followed by cold stratification (Bewley and Black 1994; Bewley 1997; Koornneef *et al.* 2002; Donohue *et al.* 2005; Finch-Savage and Leubner-Metzger 2006; Baskin and Baskin 2014).

It is well known that endogenous mechanisms, including oxidative components, are highly influenced by environmental factors. Thus, variation in oxidative stress in response to environmental factors may play a pivotal role in the breaking of seed dormancy and, subsequently, in promoting germination (Marx *et al.* 2003; Bykova *et al.* 2011). These oxidative changes are involved with reactive oxygen species (ROS), nitric oxide (NO) and the redox system (El-Maarouf-Bouteau and Bailly 2008). ROS, represented by hydrogen peroxide (H_2_O_2_), are considered to be messengers or transmitters of environmental cues during seed dormancy break, thereby promoting germination (Bailly *et al.* 2008), Their production contributes to the control of the redox status in cells (El-Maarouf Bouteau *et al.* 2007; El-Maarouf-Bouteau and Bailly 2008). Consequently, highly regulated ROS production in embryos could serve as a trigger of oxidative modification of proteins through carbonylation, thus resulting in modification of their enzymatic and binding properties, eventually leading to changes in their function (Oracz *et al.* 2007).

Although ROS in high concentrations have harmful effects, the proposed ‘oxidative window’ model suggests that highly regulated ROS generation is required for seed germination (Bailly *et al.* 2008). However, levels of ROS must be strictly controlled to fulfil their role as cellular messengers. Therefore, seeds must have an efficient antioxidant system with which to regulate ROS concentration (El-Maarouf-Bouteau and Bailly 2008). In the course of evolution, plants have developed a series of antioxidative systems that avoid ROS damage caused by environmental stress (Torres *et al.* 2000; Ahmad *et al.* 2010; Jaspers and Kangasjarvi 2010). Thus, antioxidant defences may be responsible for seed germination (De Gara *et al.* 1997; Tommasi *et al.* 2001).

Antioxidant enzymes, including superoxide dismutase (SOD), catalase (CAT) and peroxidase (POD) can scavenge excessive ROS and decrease stress-induced oxidative damage. For example, SOD is an efficient enzyme that can control intracellular concentrations of superoxide and peroxide that prevent formation of hydroxyl radicals through the Fenton reaction, thus preventing lipid peroxidation (Gutteridge and Halliwell 1990; Bowler *et al.* 1994). CAT increased in germinating sunflower seeds prior to radicle protrusion, and this response was parallel to decreases in H_2_O_2_ content and in level of lipid peroxidation (Bailly 2004). A similar increase in CAT activity has been described during germination of maize, soybean, *Arabidopsis* and sweet corn seeds (Puntarulo *et al.* 1991; Gallardo *et al.* 2001; Posmyk *et al.* 2001; Chiu *et al.* 2002).

Changes in other important antioxidant enzymes during imbibition and germination of seeds have also been documented. NO is a potent dormancy-releasing agent in many plant species, including *Arabidopsis*, and it has been suggested to serve as an endogenous regulator for physiological seed dormancy (Arc *et al.* 2013). In particular, NO can directly scavenge certain ROS such as superoxide anions and lipid-derived radicals and can stimulate antioxidant enzymes, thereby limiting oxidative damage. Therefore, NO could play a key role in germination by crosstalking with ROS. Large amounts of proline, which can serve as an organic osmoprotectant, can accumulate in plants subjected to abiotic stress (Liu *et al.* 2014). In addition to maintaining osmotic balance, proline also can stabilize subcellular structures, such as membranes and proteins, quench active oxygen and protect cells against the adverse effects of salt stress (Ashraf and Foolad 2007).

Pomegranate (*Punica granatum*), which originated in Central Asia, is an edible-fruit tree with a global geographical distribution (Jaime *et al.* 2013). It has economic, nutritional, medicinal and industrial uses (Wang *et al.* 2013). The seeds have non-deep physiological dormancy (Shalimu *et al.* 2015). In a study by Shalimu *et al.* (2015), germination percentages were greater with warm (2 months, i.e. 56 days) + cold (2 months, i.e. 56 days) stratification than with cold stratification only (3, months, i.e. 84 days). Although warm + cold stratification is an effective way to break dormancy in pomegranate seeds, no studies have been done to determine the oxidative pattern during the process of dormancy break. Considerable research has been done on internal oxidative changes during seed dormancy removal in *Arabidopsis* (Job *et al.* 2005; Graeber *et al.* 2012), sunflower (Oracz *et al.* 2007) and apple (Dębska *et al.* 2013) and on germination of seeds of sunflower and several genotypes of *Arabidopsis* after dormancy was broken (Layat *et al.* 2014). Since dormancy in mature apple seeds can be broken by cold stratification, apple seeds are accepted as a model for elucidating the endogenous oxidative changes caused by cold stratification alone (Gniazdowska et al. 2010a, b; Dębska *et al.* 2013; Krasuska *et al.* 2014).

We chose pomegranate seeds as the material with which to compare change in the oxidative pattern during warm + cold and cold stratification. We analyzed the dynamic pattern of NO, proline, H_2_O_2_, malondialdehyde (MDA) and protein carbonylation and the activities of characteristic scavenging enzymes CAT, POD and SOD in the process of dormancy break of pomegranate seeds by both warm + cold and cold stratification.

## Methods

### Seed collection

Freshly matured fruits of pomegranate cultivar Xinjiang Hotan CeLe1# were collected in Xinjiang, China, in November, 2013. The length, width and 100-seed mass of pomegranate seeds used in our study were 6.806 ± 0.047, 3.416 ± 0.032 mm and 2.55 ± 0.010 mg, respectively, and they were covered with a juicy aril that people have enjoyed eating for centuries. Seeds were separated from the fruits, washed to remove the arils and stored in cloth bags for 1 day under ambient laboratory conditions prior to use in experiments. Five groups, each with 100 seeds, were weighed individually using a Sartorious BS210S electronic balance (0.0001 g) and then placed in a drying oven set at 80 °C for 72 h. Percentage of seed moisture content (MC) was calculated as follows: MC (%) = [(fresh mass–dry mass)/fresh mass]×100. Seed MC was 7.95% ± 0.14%, and seeds were tested for viability with 2,3,5-triphenyl-2H-tetrazolium chloride (TTC). The thin tip of each seed was sliced off ∼2–3 mm carefully and placed in a 0.1% aqueous TTC solution in darkness at 20 °C for 24 h. Embryos that stained red or pink were considered to be viable and those that did not stain nonviable (Baskin and Baskin 2014).

### Stratification and measurement of germination

Stratification treatments were conducted in two steps according to Lewak and Smoleńska (1968): cold stratification only and warm + cold stratification. For the stratification treatments, 300 seeds were placed into each of 50 dishes (20 cm diameter) containing moistened sterile quartz sand. For cold stratification, seeds were kept at 4 °C in a dark environment for 1, 7, 14, 21, 42, 63 and 84 days. For warm + cold stratification, seeds were kept at 25/15 °C on a 12 h/12 h light-dark cycle for 56-days (Ws stage), and then transferred to 4 °C in the dark for 56-days (Cs stage), i.e. seeds received a total of 112 days (56 d of Ws + 56 d of Cs) of stratification. After 1 (Ws), 7 (Ws), 14 (Ws), 21 (Ws), 42 (Ws), 56 (Ws) + 7 (Cs), 56 (Ws) + 28 (Cs) and 56 (Ws) + 56 (Cs) days of stratification, seeds were tested for germination and for biochemical indicators and related enzyme activities. The viability of seeds of Cs at 84 days or Ws + Cs at 112 days was tested with TTC. Seeds with 84 days of Cs or 112 days of Ws + Cs were viable.

For germination tests, four replicates of 25 seeds were randomly selected and placed on filter paper moistened with distilled water in Petri dishes and incubated at 25/15 °C in light (12 h each day, ca. 100 µmol m^−^^2^ s^−^^1^, 400 to 700 nm, cool white fluorescent light, hereafter light) for 28 days. The seeds were checked daily and water was added as need to keep the substrate moist. A seed was considered to be germinated when the radicle had emerged (Bewley *et al.* 2013).

For examination of biochemical indicators and determination of related enzyme activities, ∼1080 seeds were removed from the sand and rinsed in tap water, and embryos were isolated from the seeds. Each seed contained a fully developed folded embryo (Martin 1946) and in our studies the whole embryo, i.e. radicle, axis and the two cotyledons, was used in experiments. Subsequently, ∼60 isolated embryos were placed on ice and used for each of the biochemical determinations.

### Examination of biochemical indicators

NO was determined colorimetrically using the Griess reagent (Bethke *et al.* 2006). Absorbance at 543 nm was performed with a Shimadzu UV-1700 spectrophotometer (Shimadzu Corp., Kyoto, Japan). NO level was assayed for six replicates (each with ∼10 embryos, total 60 embryos) and results expressed as µmol/g protein. Protein concentration was measured according to Bradford (1976) using bovine serum albumin as the standard. This method was used for measuring proteins hereafter.

Proline was determined according to Bates *et al.* (1973) and Singh *et al.* (2015), with modifications. Embryos (0.1 g, 8–10 embryos) were homogenized in 0.9 mL of 3% (w/v) sulphosalycylic acid and then centrifuged at 3500 g for 10 min to obtain the supernatant. Upon addition of 1 mL of acidic ninhydrin and 1 mL of glacial acetic acid to the supernatant, the mixture was heated at 100 °C for 30 min in a water bath, and then the reaction was terminated at room temperature (25 °C). Subsequently, this mixture was extracted with toluene, and absorbance in the toluene phase was determined at 520 nm using a Shimadzu spectrophotometer. Proline concentration was assayed for six replicates and expressed as µg/g FW.

H_2_O_2_ level in pomegranate embryos was determined according to the method that Gniazdowska *et al.* (2010a) used for apple embryos, with modifications. Briefly, 0.1 g (8–10 embryos) of embryos were homogenized in ice with 0.1% (w/v) cold trichloroacetic acid (TCA) and then centrifuged at 15 000 g for 15 min at 4 °C to collect the supernatant. The reaction mixture contained 0.5 mL supernatant, 1 mL freshly prepared 1 M KI in 10 mM potassium phosphate buffer (pH 7.0) and 0.5 mL of 10 mM potassium phosphate buffer (pH 7.0) under ambient laboratory conditions and absorbance measured at 390 nm using a Shimadzu spectrophotometer. H_2_O_2_ concentration was assayed for six replicates and expressed as µmol/g FW. 

Lipid peroxidation analysis, which is based on MDA content, followed the method of Heath and Parker (1968) and Singh *et al.* (2015), with modifications. Embryos (0.1 g, 8–10 embryos) were ground in 20 mL of 0.1% TCA solution and centrifuged at 12 000 g for 10 min to obtain supernatant. Subsequently, 1 mL of supernatant was mixed with 4 mL of 0.5% thiobarbituric acid in 20% TCA solution. The mixture was heated for 20 min at 98 °C in a water bath, and then the reaction was terminated immediately on ice. After centrifugation at 12 000 g for 10 min, absorbance was measured at 530 nm using a Shimadzu spectrophotometer. Six replicates were assessed and results expressed as nmol/g FW.

Protein carbonyl groups in embryos were examined according to the method of Levine *et al.* (1994) and Dębska *et al.* (2013), with minor modification. Embryos (0.1 g, 8–10 embryos) were homogenized in a 3 mL 0.1 M Tris-HCl (pH 7.0) buffer solution containing 1 mM EDTA, 2% (w/v) PVPP, 5 mM DTT and 1% (w/v) protease inhibitor cocktail (P9599, Sigma-Aldrich, Beijing, China). After centrifuging (15 000 g for 15 min) at 4 °C, the supernatant was filtered through cotton wool and 1% (w/v) streptomycine sulphate added. Subsequently, the supernatant was incubated in the dark for 20 min at room temperature. Aliquots of the supernatant containing 0.5 mg of protein were incubated at 37 °C for 35 min in the dark with 500-µL of 10 mM 2,4-dinitrophenylhydrazine (DNPH) (42210, Sigma-Aldrich, Beijing, China) in 2 M HCl. The same supernatant incubated in a 500-µL of 2 M HCl without DNPH served as the blank. Proteins were precipitated with 500 µL of 20% TCA for 10 min, and then the pellets were washed three times with a mixture of ethanol and ethyl acetate (1:1, v/v). Following each wash, samples were centrifuged for 5 min at 10 000 g to ensure purification. Pellets were then dissolved in 6 M guanidine hydrochloride (G4505, Sigma-Aldrich, Beijing, China) in 2 M HCl. Absorbance was measured with the Shimadzu spectrophotometer at 375 nm, and the concentration of protein carbonyl groups was calculated from the extinction coefficient for DNPH (ϵ = 22 000 M^−^^1^ cm^−^^1^). There were six replicates for each group, and results expressed as nmol/g protein.

### Determination of enzyme activities

Activity of SOD was determined by measuring its ability to inhibit photochemical reduction of nitroblue tetrazolium (NBT) according to the method of Giannopolitis and Ries (1977) and Puyang *et al.* (2015). Embryos (0.1 g, 8–10 embryos) were homogenized in light in 3 mL of a reaction mixture containing 63 µM NBT, 1.3 µM riboflavin, 13 mM methionine, 0.1 mM EDTA, 50 mM phosphate buffer (pH 7.8) and 30 µL enzyme extract. The reaction was initiated by switching on the light and was allowed to run for 10 min. The reaction was terminated by switching off the light, and then absorbance at 560 nm was measured using the Shimadzu spectrophotometer. A non-illuminated reaction mixture that did not develop colour served as control, and activity of SOD was calculated by subtracting A_560_ (control) from A_560_ (SOD). One unit (U) of SOD activity was defined as the amount of enzyme required to cause 50% inhibition of the NBT reduction rate. The activity of SOD was assayed for six replicates and expressed as U/g FW.

Activities of CAT and POD were measured using the method of Chance and Maehly (1955) and Zhang and Kirkham (1996). For CAT, the H_2_O_2_ decomposition rate was determined by calculating the time elapsed during the decline in absorbance at 240 nm using the Shimadzu spectrophotometer. Embryos were homogenized in 3 mL of a reaction mixture containing 50 mM phosphate buffer (pH 7.0), 15 mM H_2_O_2_ and 0.1 mL enzyme extract. The reaction was initiated with enzyme extract. Milli-Q water without enzyme was the control, and activity of CAT was calculated by subtracting A_240_ (CAT) from A_240_ (control). For POD, the oxidation rate of guaiacol was measured by calculating the increase in absorbance at 470 nm using the Shimadzu spectrophotometer. The assay contained 50 µL of 20 mM guaiacol, 2.83 mL of 10 mM phosphate buffer (pH 7.0) and 0.1 mL enzyme extract. The reaction was initiated by adding 20 µl of 40 mM H_2_O_2_. Milli-Q water without enzyme was the control, and activity of POD was calculated by subtracting A_240_ (control) from A_240_ (POD). CAT and POD activities were assayed for six replicates and expressed as U/mg protein.

### Statistical analysis

Data were analyzed using SPSS for Windows (Version 17.0, SPSS Inc., Chicago, IL, USA). All data were analyzed for normality and homogeneity of variance prior to analysis, and if normal and homogeneous they were subjected to further analysis. If data were not normally distributed or if variances were not homogeneous, they were log_10_ transformed before analysis to ensure homogeneity of variance. In cases where the ANOVA assumptions continued to be violated following data transformation, treatment differences were assessed using the more conservative Kruskal–Wallis nonparametric test. Tukey's honestly significant difference (Tukey's HSD test) was used to test for differences (*P* <0.05) between warm + cold stratification and cold stratification only. Statistical tests were conducted at *P* = 0.05. Values are means ± s.d. (Sokal and Rohlf 1995). 

When variances of data were homogeneous, one-way ANOVA was used to determine differences among germination percentages, NO, proline, H_2_O_2_, MDA, protein carbonylation and activities of the ROS scavenging enzymes SOD, CAT and POD in embryos during warm + cold stratification and cold stratification only. Two-way ANOVA was used to test the significance of main effects (stratification treatment and stratification period) and their interaction with germination, H_2_O_2_, MDA, protein carbonylation, SOD, hydrogen peroxide enzyme, POD, NO and proline. The correlations between different assays were calculated using the correlation coefficient statistical option in the Pearson test. Correlation analysis was used to determine the relationship between NO, H_2_O_2_, proline and germination percentages; the relationship between MDA, protein carbonylation and H_2_O_2_; and the relationship between H_2_O_2_ and the ROS scavenging enzymes SOD, CAT and POD.

## Results

### Effects of stratification treatment and stratification period

Two-way ANOVAs showed that germination, NO level, concentration of proline, MDA and protein carbonyl groups, activity of CAT and activity of POD were significantly affected by stratification treatment (warm + cold stratification and cold stratification only) and stratification period (for corresponding df*, F* and *P* values, see Table 1). Also, there were significant interactions between stratification treatment and stratification period (Table 1). However, two-way ANOVA indicated that activity of SOD and H_2_O_2_ level were significantly affected by stratification period instead of stratification treatment. Nevertheless, there were still significant interactions between stratification treatment and stratification period in the H_2_O_2_ level and activity of SOD assays (Table 1).

**Table 1.** Two-way ANOVA of effects of stratification treatment (warm + cold stratification and cold stratification only), stratification period and their interactions on oxidative parameters in pomegranate seeds during warm + cold stratification and cold stratification only. CAT, catalase; PC, protein carbonylation; Pro, proline.

	Source	Degrees of freedom	Sum of squares	Mean of squares	*F*-value	*P*-value
GP	Stratification treatment (T)	1	5620.018	5620.018	1237.435	<0.01
	Stratification period (P)	6	18508.107	3084.685	679.197	<0.01
	T × P	6	1870.107	311.685	68.628	<0.01
H_2_O_2_	T	1	4.286E-5	4.286E-5	56.513	<0.01
	P	6	0.094	0.016	0.154	0.696
	T × P	6	0.017	0.003	10.139	<0.01
MDA	T	1	7.324	7.324	3935.833	<0.01
	P	6	3.427	0.571	306.982	<0.01
	T × P	6	3.082	0.514	276.007	<0.01
PC	T	1	0.099	0.099	97.511	<0.01
	P	6	2.101	0.350	346.320	<0.01
	T × P	6	0.378	0.063	62.310	<0.01
SOD	T	1	0.004	0.004	2.470	0.121
	P	6	0.338	0.056	31.741	<0.01
	T × P	6	0.049	0.008	4.601	<0.01
CAT	T	1	0.100	0.100	30.863	<0.01
	P	6	0.482	0.080	24.712	<0.01
	T × P	6	0.691	0.115	35.419	<0.01
POD	T	1	0.158	0.158	89.621	<0.01
	P	6	0.138	0.023	13.074	<0.01
	T × P	6	0.082	0.014	7.755	<0.01
NO	T	1	0.000	0.000	23.170	<0.01
	P	6	0.036	0.006	362.060	<0.01
	T × P	6	0.052	0.009	517.637	<0.01
Pro	T	1	2.380	2.380	1725.903	<0.01
	P	6	6.455	1.076	780.128	<0.01
	T × P	6	0.344	0.057	41.549	<0.01

### Warm + cold stratification alleviation of dormancy of pomegranate seeds

**Figure 1.**
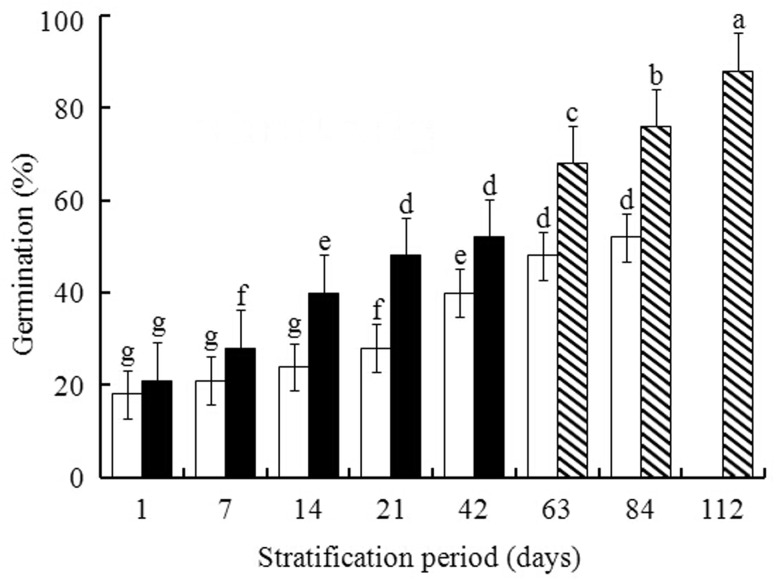
Effect of warm + cold [warm 1–42 days (closed bar), warm 56 days + cold 7, 28 or 56 days (striped bar)] and cold (open bar) stratification on germination percentages (mean ± s.d.) of pomegranate seeds. Different lowercase letters in a bar indicate significant difference (Tukey’s HSD, *P* = 0.05).

Warm + cold stratification had a positive effect on breaking seed dormancy. In warm + cold stratification, germination increased from 20% to 85% for seeds stratified for 56 Ws + 56 Cs. However, germination of seeds cold stratified for 84 days increased from 19% to only 50% (Fig. 1). Seed cold-stratified for 112 days were dead, as shown by the TTC test (data not shown).

### Dormancy alleviation by warm + cold stratification involved changes in NO

**Figure 2.**
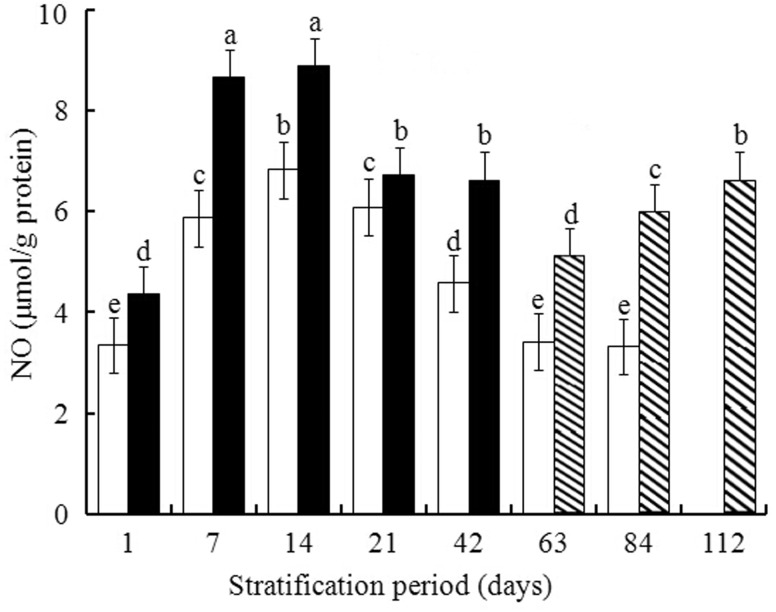
Effect of warm + cold [warm 1–42 days (closed bar), warm 56 days + cold 7, 28 or 56 days (striped bar)] and cold (open bar) stratification on production of NO in embryos isolated from pomegranate seeds. Values are mean ± s.d. Different lowercase letters in a bar indicate significant difference (Tukey’s HSD, *P* = 0.05).

For warm + cold stratification, NO level at Day 7 was 8.66 ± 0.17 µmol/g protein, and then it increased slightly to 8.78 ± 0.33 µmol/g protein at Day 14. After 14 days, NO declined rapidly until Day 63 and then rebounded at Day 84, reaching a second peak (6.28 ± 0.26 µmol/g·protein) at Day 112 (Fig. 2). During cold stratification, NO levels increased gradually reaching a short-term maximum (∼6.81 ± 0.18 µmol/g·protein) at 2 weeks of treatment. After 14 days of cold treatment, NO levels had decreased to the initial level at Day 84 (Fig. 2).

### Dormancy alleviation by warm + cold stratification was accompanied by increased proline concentration

**Figure 3.**
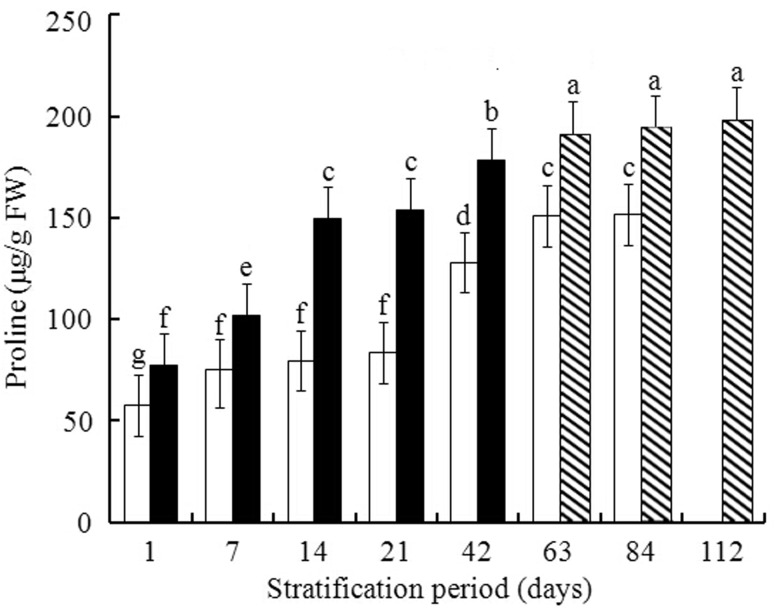
Effect of warm + cold [warm 1–42 days (closed bar), warm 56 days + cold 7, 28 or 56 days (striped bar)] and cold (open bar) stratification on concentration of proline in embryos isolated from pomegranate seeds. Values are mean ±  s.d. Different lowercase letters in a bar indicate significant difference (Tukey’s HSD, *P* = 0.05).

Proline concentration increased steadily between 1 and 63 days of warm + cold stratification and did not significantly increase after that (Fig. 3). For cold stratification, proline concentration increased between 1 and 7 days, did not significantly change between 7 and 21 days of stratification, but then increased, also reaching a maximum at 63 days. At each assessment time, proline concentration for cold stratification seeds was significantly lower than that for warm + cold stratification seeds (Fig. 3).

### Warm + cold stratification influenced levels of H_2_O_2_, MDA and protein carbonylation

**Figure 4.**
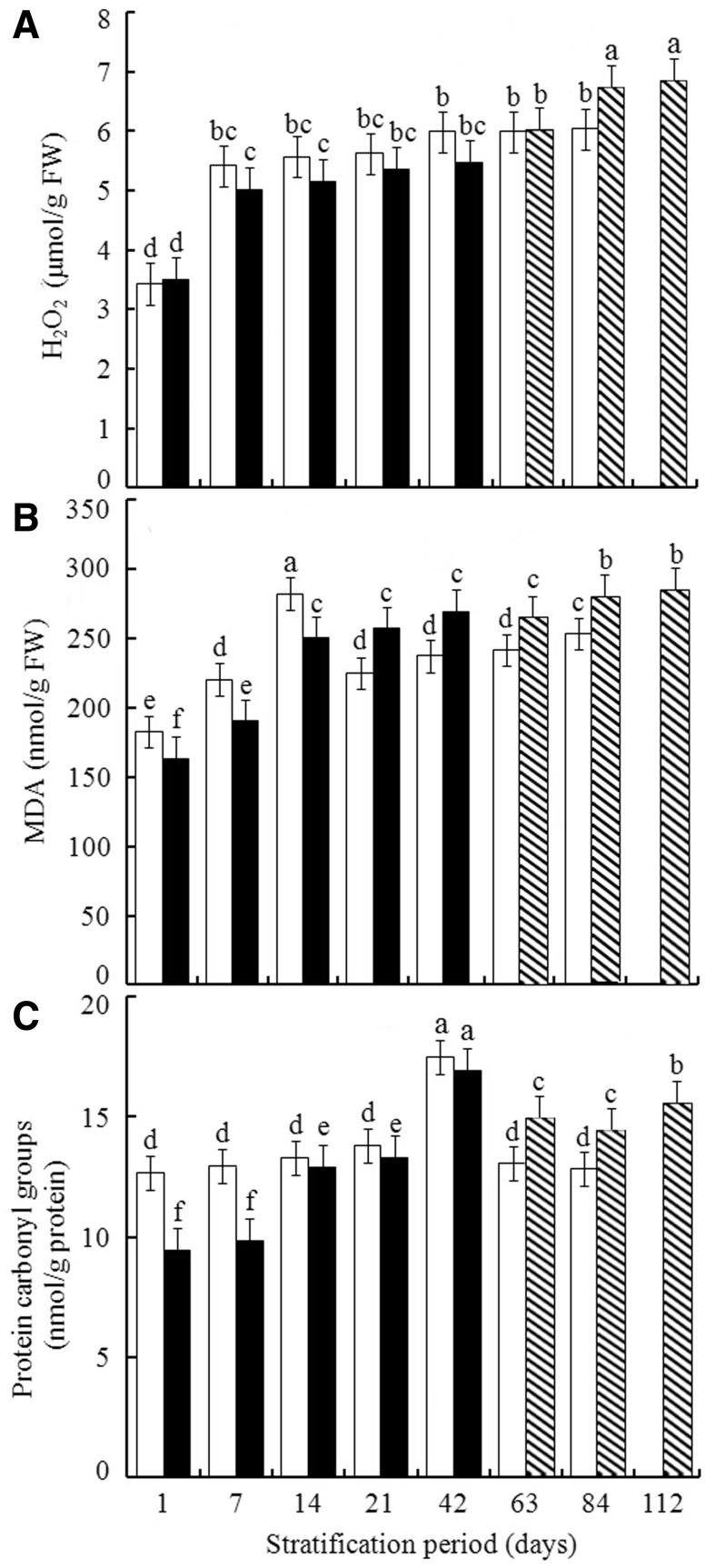
Effect of warm + cold [warm 1–42 days (closed bar), warm 56 days + cold 7, 28 or 56 days (striped bar)] and cold (open bar) stratification on concentration of hydrogen peroxide (H_2_O_2_), MDA and protein carbonyl groups in embryos isolated from pomegranate seeds. Values are the mean ± s.d. Different lowercase letters in a bar indicate significant difference (Tukey’s HSD, *P* = 0.05).

Concentration of H_2_O_2_ increased to ∼5.3 ± 0.39 µmol/g FW at Day 7 of cold stratification and did not significantly increase between 7 and 21 days or between 42 and 84 days, but there was a significant increase between 21 and 42 days (Fig. 4A). In the warm + cold stratification, H_2_O_2_ concentration increased significantly between 1 and 7 days, did not change significantly between 7 and 42 days, and reached the maximum at 84 day, with no significant difference between 84 and 112 days.

At the beginning of the cold stratification treatment (1–14 days), MDA concentration in pomegranate embryos increased from 190.69 ± 12.65 nmol/g FW to 282.35 ± 35.42 nmol/g FW. It decreased significantly between 14 and 21 days but did not increase significantly between 21 and 63 days. It reached a maximum at 84 days (Fig. 4B). For warm + cold stratification, MDA increased between Days 1 and 14, did not differ significantly between Days 14 and 63, then further increased at Day 84, with no difference between Days 84 and 112. Different periods of cold stratification did not result in significant differences in the level of protein carbonylation. The carbonylation levels of warm + cold stratification seeds did not differ significantly between 1 and 7, 14 and 21, and 63 and 112 days, with average values of 9.29 ± 2.88, 13.66 ± 1.67 and 15.11 ± 3.12 nmol/g protein, respectively. Furthermore, the carbonylation levels of 42 days of both cold (17.48 ± 3.85 nmol/g protein) and warm + cold (16.78 ± 4.11 nmol/g protein) stratifications were the highest ones.

### Warm + cold stratification influenced activities of ROS scavenging enzymes

**Figure 5.**
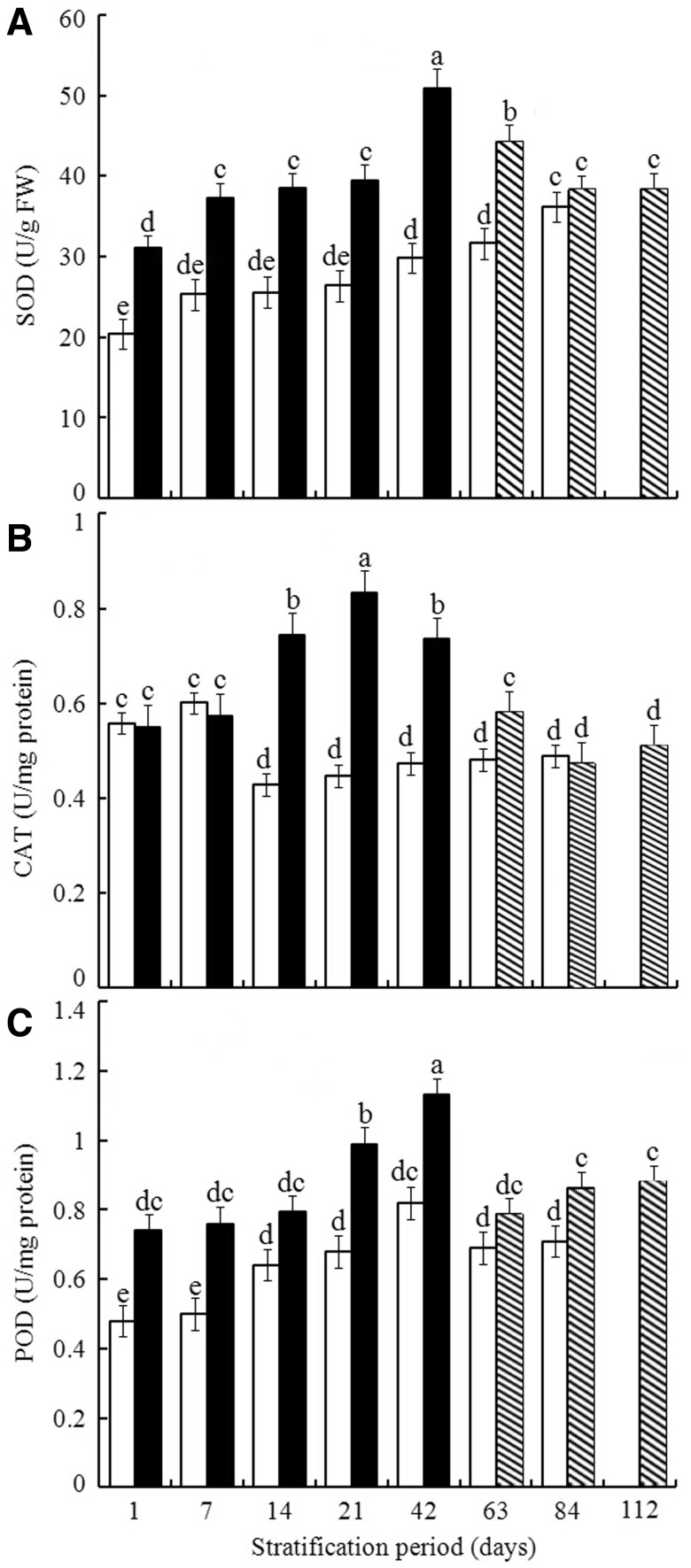
Effect of warm + cold [warm 1–42 days (closed bar), warm 56 days + cold 7, 28 or 56 days (striped bar)] and cold (open bar) stratification on changes in activity of SOD, CAT and POD in embryos isolated from pomegranate seeds. Values are mean ±  s.d. Different lowercase letters in a bar indicate significant difference (Tukey’s HSD, *P* = 0.05).

In embryos isolated from cold stratified seeds, the activity of SOD did not differ between Days 1–21, or between Days 7 and 63, but it was significantly greater at Day 84 (Fig. 5A). The activity of SOD was 32.43 ± 0.64 U/g FW after the first day of warm + cold stratification. There was a significant increase between Day 1 and Day 7, no significant change between Days 7 and 21, a significant increase at Day 42, and a decrease after that. Under prolonged warm + cold treatment, the SOD activity declined to a level of ∼ 38.41 ± 1.34 U/g FW at Day 84 (Fig. 5A).

For cold stratification, activity of CAT fell after Day 7 and did not change significantly for the rest of the time. For warm + cold stratification, the activity of CAT increased up to 21 days and decreased after that time (Fig. 5B). For cold stratification, POD activity did not differ significantly between Days 1 and 7, increased significantly up to Day 42, but it did not change significantly for all the other assessments. For warm + cold stratification, POD activity did not change significantly between Days 1 and 14, increased significantly up to Day 42, and decreased significantly between Days 42 and 63. The activity increased slightly after Day 63, and it remained at a similar level between Days 84 and 112 (Fig. 5C).

## Discussion

This study clearly demonstrated that warm + cold stratification had a stronger dormancy-breaking effect than cold stratification alone on pomegranate seeds, and it established that there is a different pattern of oxidation changes in seeds receiving the two sequential treatments. To our knowledge, this is the first study on the oxidation changes occurring during dormancy-breaking via warm + cold stratification. Shalimu *et al.* (2015) showed that pomegranate seeds exhibit non-deep physiological dormancy and can germinate to 75–80% after 1–3 months warm stratification followed by 2 months cold stratification. Further, germination percentages after warm + cold stratification were greater than those after 3 months of cold stratification.

Our results in Fig. 4A showed that the H_2_O_2_ concentrations in seeds following cold stratification or warm + cold stratification did not differ significantly, although the germination percentages of warm + cold stratified were significantly greater than those of cold stratified (Fig. 1). On the other hand, the activities of ROS scavenging enzymes, including SOD, CAT and POD of warm + cold stratification were significantly greater than those of cold stratification (Fig. 5). The results indicates that H_2_O_2_ concentration in seeds with warm + cold stratification might be attenuated by the higher activities of the ROS scavenging enzymes mentioned above, leading to the similar levels of H_2_O_2_ to those in the seeds with cold stratification. The concentrations of MDA and protein carbonylation are mutually correlated (Oracz *et al.* 2007; Graeber *et al.* 2012), which might also be responsible for the similarities of their concentrations between cold and warm + cold stratification (Fig. 4B and C).

Recent reports have highlighted that, in addition to ROS, NO plays an essential role in the regulation of various developmental processes including dormancy breaking (Matakiadis *et al.* 2009; Kranner *et al.* 2010; Diaz-Vivancos *et al.* 2013), and it accumulates during seed germination in various species including *Arabidopsis* and apple (Caro and Puntarulo 1999; Sarath *et al.* 2007; Dębska *et al.* 2013). We found a pattern of increasing NO that was relatively stronger during warm + cold stratification than during cold stratification only (Fig. 2), which might contribute to the greater germination percentage in seeds following warm + cold stratification (Fig. 1). However, this speculation was not supported by the data shown in Table 2, in which NO was not correlated with germination for either of the two treatments. This contradiction need to be investigated further.

**Table 2.** Correlation coefficients between germination percentage and oxidative parameters in pomegranate seeds during warm + cold stratification (below diagonal line) and cold stratification (above diagonal line). GP, germination percentage; PC, protein carbonylation; CAT, catalase; Pro, proline. *P <* *0.05. **P <* *0.01.

	GP	H_2_O_2_	MDA	PC	SOD	CAT	POD	NO	Pro
GP	1	0.692	0.790*	0.980*	0.946**	−0.373	0.746	−0.548	0.995**
H_2_O_2_	0.785*	1	0.982**	0.645	0.787*	−0.503	0.754	0.198	0.711
MDA	0.697	0.903**	1	0.739	0.887**	−0.463	0.748	0.051	0.795*
PC	0.734*	0.705	0.833*	1	0.904**	−0.338	0.666	−0.586	0.982**
SOD	0.353	0.344	0.521	0.764*	1	−0.343	0.665	−0.362	0.924**
CAT	−0.345	0.140	0.372	0.284	0.382	1	−0.707	−0.256	−0.406
POD	0.252	0.387	0.563	0.777*	0.758*	0.576	1	−0.093	0.778*
NO	−0.465	−0.006	0.062	−0.252	0.138	0.561	0.073	1	−524
Pro	0.929**	0.825*	0.851**	0.877**	0.616	−0.064	0.461	−0.262	1

Proline, as an organic osmoprotectant, accumulates in vacuoles and facilitates maintenance of water potential and water absorption by plant cells. In addition to maintaining osmotic balance, proline is also capable of stabilizing subcellular structures, quenching active oxygen and protecting cell integrity (Ashraf and Foolad 2007). Exogenous proline may improve germination percentages of different crops (Athar *et al.* 2009; Nawaz *et al.* 2010). Here, we demonstrated that there is a relatively stronger elevation of proline concentration in the embryo during warm + cold stratification than during cold stratification only, implying that proline contributed to dormancy breaking in the pomegranate seeds.

It is well-established that, depending on its concentration, ROS have positive effects on breaking seed dormancy and promoting germination via the ROS signalling pathway (Liu *et al.* 2010). H_2_O_2_, as an indicator of ROS, is continuously produced as a byproduct of various metabolic pathways (Diaz-Vivancos *et al.* 2013). Many studies have documented that there is a dynamic pattern of change in H_2_O_2_ concentration during seed germination in species such as *Arabidopsis*, sunflower and apple (Gidrol *et al.* 1994; Caliskan and Cuming 1998; Hite *et al.* 1999; Schopfer *et al.* 2001; Bailly *et al.* 2002; Morohashi 2002; Wojtyla *et al.* 2006).

ROS production can subsequently lead to lipid peroxidation, which is usually determined by MDA formation (Oracz *et al.* 2007; Ahmad *et al.* 2010). Lipid peroxidation weakens cell membranes and thus may increase cell membrane permeability, thereby enhancing seed imbibition. The MDA concentrations in seeds following cold stratification were significantly greater than those of warm + cold stratified seeds during the early stage of the two treatments from Day 1 to 14, whereas the relative concentrations of the two treatments were opposite during the late stage from Day 21 to Day 84 in these two treatments (Fig. 4B). This might be a possible reason for the discrepancy in the correlation between germination percentage and MDA concentration in the seeds following the two different treatments, with cold stratification being significantly correlated with MDA concentration but not so for warm + cold stratification (Table 2).

Proteins are the downstream target of ROS during metabolic changes that occur during dormancy-break in seeds (Oracz *et al.* 2007; Tanou *et al.* 2009; Dębska *et al.* 2013), and protein carbonylation is an irreversible oxidative process that causes a loss of protein function (Diaz-Vivancos *et al.* 2013). However, carbonylation in seeds is not necessarily a negative response and may promote dormancy-break and subsequent germination (Job *et al.* 2005; Rajjou *et al.* 2006; Oracz *et al.* 2007; Arc *et al.* 2013). Further, carbonylation of storage proteins may contribute to both mobilization of reserves and *de novo* synthesis of proteins that are required for germination. Therefore, protein carbonylation can play a pivotal role in seed dormancy break and germination (Barba-Espín *et al.* 2011; Diaz-Vivancos *et al.* 2013).

Since ROS must be at a proper level to function properly as cellular messengers, effective antioxidant systems must be present to regulate the level of ROS (El-Maarouf-Bouteau and Bailly 2008). The antioxidant control systems involving the enzymes SOD, CAT and POD make an important contribution to the transition from dormancy to germination (De Gara *et al.* 1997; Tommasi *et al.* 2001). Warm + cold stratification-induced ROS accumulation in pomegranate seeds led to an elevation followed by attenuation of the activities of the scavenging enzyme system, including SOD, CAT and POD, and thus to changes in the pattern of ROS activity. These results indicate that the strong activity of ROS scavenging enzymes at the beginning of this stratification procedure effectively restricted H_2_O_2_ concentration to a lower level, thus leading to a low germination percentage. There was no relationship between activity of ROS scavenging enzymes and germination percentage for either treatment (Table 2). This might be due to the fact that the activities of the ROS scavenging enzymes in the two treatments had both reached maxima at Day 21 or 42 and then declined (Fig. 5). The results also indicated that increases in the activities of the enzymes at the early stage of the two treatments might contribute largely to the increases in germination percentages. On the other hand, changes in the activities of these three scavenging enzymes during cold stratification only were generally lower than those during warm + cold stratification.

Correlations analysis revealed that H_2_O_2_ was significantly positively correlated with germination percentage in the warm + cold stratification treatment (*r *= 0.785, *P *= 0.021), whereas H_2_O_2_ was not significantly positively correlated with germination percentage in cold stratification treatment (*r *= 0.692, *P *= 0.085) (Table 2). These data may to some degree account for high germination percentages of seeds that were given warm + cold stratification. Further, there were significant positive correlations between H_2_O_2_ and MDA in warm + cold stratified seeds (*r *= 0.903, *P *= 0.002) and cold stratified only treatments (*r *= 0.982, *P *< 0.01), suggesting that accumulation of H_2_O_2_ induced lipid peroxidation in both stratification treatments. Moreover, MDA was significantly positively correlated with protein carbonylation during warm + cold stratification (*r *= 0.833, *P *= 0.010) but not during cold stratification (*r *= 0.739, *P *= 0.122), suggesting that protein carbonylation was closely related to the accumulation of lipid peroxidation in the warm + cold stratification treatment.

There are positive correlations between protein carbonylation and germination percentage in both stratification treatments but not between NO and germination percentage during warm + cold (*r *= −0.465, *P *= 0.246) or cold stratification (*r *= −0.0548, *P *= 0.203). These results suggest that NO may serve only as a signal regulator and that it is not directly involved in seed germination (Arc *et al.* 2013). Germination percentages were significantly positively correlated with proline concentration during warm + cold stratification (*r *= 0.995, *P *< 0.01) and during cold stratification (*r *= 0.929, *P *= 0.001), which suggest that proline may effectively improve germination of pomegranate seeds.

Activity of the scavenging enzyme SOD was significantly positively correlated with H_2_O_2_ (*r *= 0.787, *P *= 0.036) during cold stratification but not during warm + cold stratification (*r *= 0.344, *P *= 0.405). Neither the activity of CAT nor POD was significantly correlated with H_2_O_2_ either during warm + cold stratification (*r *= −0.503, *P *= 0.250; *r *= 0.754, *P *= 0.050) or cold stratification (*r *= 0.140, *P *= 0.742; *r *= 0.387, *P *= 0.343). This lack of correlation might be attributed to the nonparallel variation (increase followed by decrease) between ROS scavenging enzyme activity during warm + cold stratification and cold stratification only, which is not completely the same pattern as that of H_2_O_2_. The lack of correlation was further supported by the irregular pattern of activities of the ROS scavenging enzymes, which were increased up to Day 21, then either declined after 42 (Fig. 5A) and Day 21 (Fig. 5B), or did not differ after Day 42 (Fig. 5C). Figure 5 clearly shows that the activities of ROS scavenging enzymes during both stratification treatments decreased after either 21 (Fig. 5B) or 42 (Fig. 5A and C) days, being consistent with the increasing pattern of H_2_O_2_ at about the same stage (Fig. 4A).

## Conclusions

**Figure 6.**
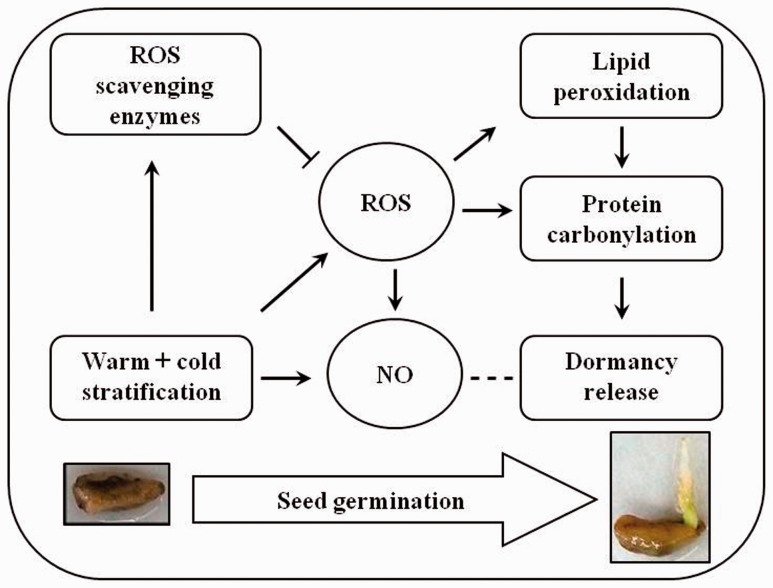
A conceptual model for mechanism of dormancy break in pomegranate seeds by warm + cold stratification. Note: solid line means direct influence and dashed line means indirect influence.

A conceptual model for the mechanism of dormancy break by warm + cold stratification is proposed in Figure 6. At the beginning (1–14 days) of both warm + cold stratification and cold stratification alone, there were parallel increasing trend in the content of NO (Fig. 2), H_2_O_2_ (Fig. 4A), and lipid peroxidation (Fig. 4B). Later in the warm + cold treatment, even in the terminal phase of the cold stratification portion of the treatment, the level of NO, H_2_O_2_, lipid peroxidation and protein carbonyl groups was high. However, levels of ROS must be strictly controlled to fulfil their role as cellular messengers. Therefore, ROS scavenging enzymes (SOD, CAT and POD) can effectively regulate the concentration of ROS thus avoiding damage to ROS. In addition, NO can directly scavenge certain ROS, such as superoxide anions and lipid-derived radicals, and stimulate antioxidant enzymes, thereby limiting oxidative damages. Therefore, we suggest that dormancy break and subsequent germination in pomegranate seed during warm + cold stratification is correlated with the overall pattern of changes in NO, ROS, lipid oxidation and protein carbonylation.

## Sources of Funding

This work was supported by the Fundamental Research Funds for the Central Universities (Nos. BLYJ201413).

## Contributions by the Authors

Y.L., D.S. and L.S. conceived and designed research. D.S. and J.S. (protein measurements) conducted experiments. D.S., C.C.B. and J.M.B. analyzed data. D.S., Y.L. and L.S. wrote the manuscript. All authors read and approved the manuscript.

## Conflict of Interest Statement

None declared.
